# Motor Learning Unfolds over Different Timescales in Distinct Neural Systems

**DOI:** 10.1371/journal.pbio.1002313

**Published:** 2015-12-08

**Authors:** Janelle Weaver

**Affiliations:** Freelance Science Writer, Carbondale, Colorado, United States of America

## Abstract

A new study reveals the time-resolved brain map of activity involved in the formation of motor memories, from seconds to hours and from the frontal and parietal lobes to the cerebellum. Read the Research Article.

Anyone who has learned how to play a musical instrument knows that translating notes on a sheet into finger movements is effortful at first, but gradually becomes more automatic over time. This widely appreciated feature of motor learning was described in 1967 by Paul Fitts and Michael Posner. In a book entitled *Human Performance*, the well-known psychologists proposed three stages of learning motor skills: a cognitive phase, an associative phase, and an autonomous phase.

In the first stage, movements are slow, inconsistent, and inefficient, and large parts of the movement are controlled consciously. In the second stage, movements become more fluid, reliable, and efficient, and some parts of the movement are controlled automatically. And in the third stage, movements are accurate, consistent, and efficient, and movement is largely controlled automatically. However, it has not been clear exactly how the different stages of motor learning map onto neural systems in the brain.

In a study published in this issue of *PLOS Biology*, Nicolas Schweighofer of the University of Southern California and Hiroshi Imamizu of the University of Tokyo combined computational modeling with behavioral and functional magnetic resonance imaging (fMRI) data to create a brain-wide map of motor memories with different timescales (Fig 1). According to the authors, the findings shed new light on a classical psychological theory and could potentially be used to improve strategies for the rehabilitation of motor skills after brain damage.

**Fig 1:**
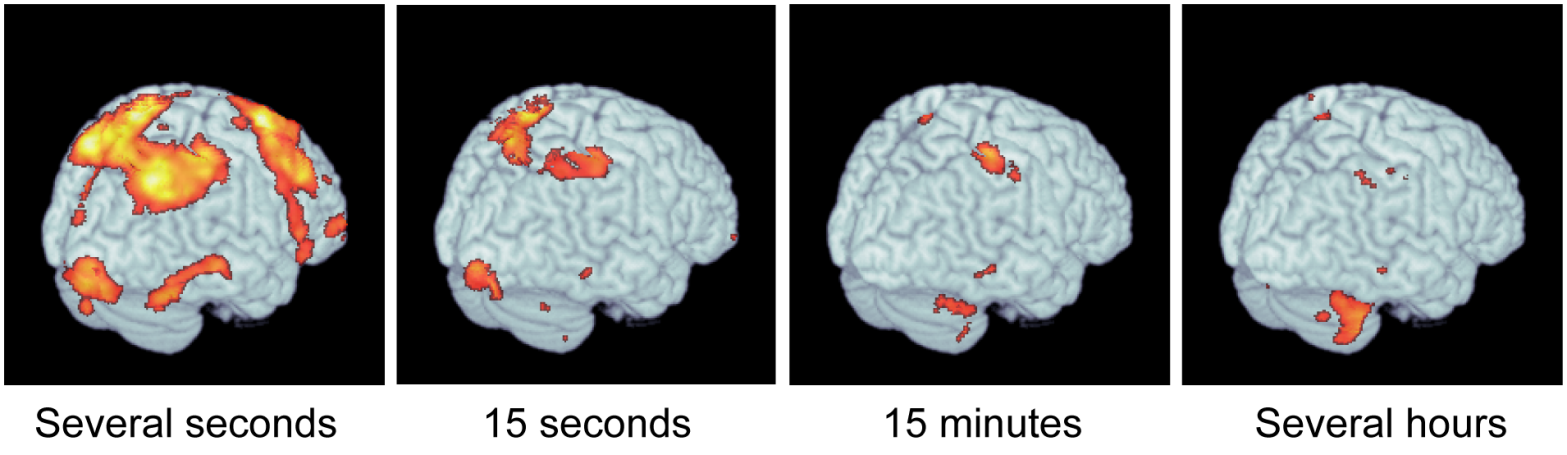
Different timescales for motor learning. Learning takes place in different brain regions at different timescales. The four panels in the figure represent time constants that characterize the speed of changes. *Image credit*: *Hiroshi Imamizu*.

In the new study, 21 healthy volunteers performed visual-motor adaptation tasks while their brain activity was measured with fMRI. At the beginning of each trial, a white cross (cursor) appeared at the center of the screen, and subjects then manipulated a joystick to move the cursor to a red or blue circle that appeared at the top of the screen. But there was a visual-motor mismatch: The cursor was rotated by 40 degrees relative to the actual movement direction. Over time, the subjects learned to adapt to this rotation by adjusting the joystick movement in the opposite direction.

The behavioral data revealed multiple stages of motor learning, with fast adaptation occurring within blocks of nine trials and slow adaptation occurring across the blocks. The researchers then developed a model to determine which neural systems were involved in the different stages of motor learning. They found that fast learning taking place within five seconds was associated with activity in frontal and parietal brain regions. By contrast, intermediate learning between two minutes and about one and a half hours was associated with activity in the anterior region of the inferior parietal lobe. The slowest learning stage, which unfolded over hours, was associated with activity in the anterior to medial portions of the cerebellum—a brain region that plays an important role in motor control.

These findings are consistent with past research showing that frontal regions are involved in early learning stages of attention, arousal, visual motion analysis, spatial working memory, memory of hand movements, and movement planning. Similarly, parietal regions are known to play a role in early learning stages of mental and visual-motor rotation. Taken together, the findings suggest that the initial cognitive and associative phases of motor learning recruit frontal and parietal brain regions, whereas the late stage of autonomous learning depends on the anterior-medial cerebellum. Thus, by combining multiple complementary techniques, the researchers provided deeper insights into a classical and influential psychological theory proposed several decades ago.
